# How can we evaluate the potential of innovative vaccine products and technologies in resource constrained settings? A total systems effectiveness (TSE) approach to decision-making

**DOI:** 10.1016/j.jvacx.2020.100078

**Published:** 2020-10-06

**Authors:** Siobhan Botwright, Anna-Lea Kahn, Raymond Hutubessy, Patrick Lydon, Joseph Biey, Abdoul Karim Sidibe, Ibrahima Diarra, Mardiati Nadjib, Auliya A. Suwantika, Ery Setiawan, Rachel Archer, Debra Kristensen, Marion Menozzi-Arnaud, Ado Mpia Bwaka, Jason M. Mwenda, Birgitte K. Giersing

**Affiliations:** a Department of Immunization, Vaccines & Biologicals, World Health Organization Headquarters, 20 Avenue Appia, 1211-CH 27 Geneva, Switzerland; b Inter-Country Support Team, Regional Office for Africa, World Health Organization, Ouagadougou, Burkina Faso; c WHO Country Office for Mali (OMS/MALI), Quartier Ntomiboro-Bougou, B.P. 99, Bamako, Mali; d Direction Générale de la Santé et de l’Hygiène Publique, Cité Administrative Bamako, Bamako BP 232, Mali; e Health Financing Activity, United States Agency for International Development (USAID), Daerah Khusus Ibukota Jakarta 10110, Indonesia; f Department of Pharmacology and Clinical Pharmacy, Universitas Padjadjaran, Indonesia Jl. Raya Bandung-Sumedang Km. 21 Jatinangor, Sumedang, West Java 45363, Indonesia; g Center of Excellence in Higher Education for Pharmaceutical Care Innovation, Universitas Padjadjaran, Indonesia Jl. Raya Bandung-Sumedang Km. 21 Jatinangor, Sumedang, West Java 45363, Indonesia; h Health Intervention and Technology Assessment Program (HITAP), Ministry of Public Health, 6th Floor, 6th Building, Department of Health, Ministry of Public Health, Tiwanon Road, Muang, Nonthaburi 11000, Thailand; i PATH, Rue de Varembé 7, 1202 Geneva, Switzerland; j Gavi, the Vaccine Alliance, Global Health Campus, Chemin du Pommier 40, 1218 Grand, Saconnex, Geneva, Switzerland; k Regional Office for Africa, World Health Organization, Brazzaville, Congo

**Keywords:** R&D, Vaccine, Delivery technology, MCDA, Immunisation, Prioritisation

## Abstract

Innovations in vaccine product attributes could play an important role in addressing coverage and equity (C&E) gaps, but there is currently a poor understanding of the full system impact and trade-offs associated with investing in such technologies, both from the perspective of national immunisation programmes (NIPs) and vaccine developers. Total Systems Effectiveness (TSE) was developed as an approach to evaluate vaccines with different product attributes from a systems perspective, in order to analyse and compare the value of innovative vaccine products in different settings.

The TSE approach has been advanced over the years by various stakeholders including the Bill and Melinda Gates Foundation (BMGF), Gavi, PATH, UNICEF and WHO. WHO further developed the TSE approach to incorporate the country perspective into immunisation decision-making, in order for countries to evaluate innovative products for introduction and product switch decisions, and for vaccine development stakeholders to conduct their assessments of product value in line with country preferences. This paper describes the original TSE approach, development of the tool and processes for NIPs to apply the WHO TSE approach, and results from piloting in 12 countries across Africa, Asia and the Americas. The WHO TSE framework emerged from this piloting effort.

The WHO TSE approach has been welcomed by NIP and vaccine development stakeholders as a useful tool to evaluate trade-offs between different products. It was emphasised that the concept of “total systems effectiveness” is likely to be context-specific and that TSE is valuable in facilitating a deliberative process to articulate NIP priorities, for decisions around product choice, and for prioritising the development of future vaccine innovations.

## 1 Context

Globally, less than 20 per cent of children are fully vaccinated with all the globally recommended vaccines by the age of five [1]. Innovations in vaccine product attributes have the potential to reduce or remove bottlenecks to achieving global vaccine coverage and equity, by addressing logistical delivery issues and improving programme efficiency. However, many innovative products are not accessible to low-and-middle-income country (LMIC) markets: if they do become available, they are often not considered suitable for use by national immunisation programmes, or are under-utilised. Given the diverse immunisation programme needs within LMICs, and the likelihood that innovative products will cost more than existing vaccines, many innovative products may only be used to target hard to reach or unvaccinated populations. This sub-national implementation strategy places significant uncertainty on the size of demand and influences whether products can be manufactured at a sufficient scale to offer an affordable price to countries. In addition, it is often unclear whether LMICs would be willing to pay a higher procurement cost for vaccine products with features that facilitate vaccine delivery or administration to under- or unimmunised populations. As a result, manufacturers may be reluctant to invest in the development of such products, as illustrated by the slow development of microarray patches, for vaccines that are delivered through LMIC immunisation programmes (Table 1). Tools that enable a holistic assessment to compare the relative merits and drawbacks of vaccine product innovations, with consideration of equity, have not commonly been available to inform investment in novel vaccine products. This concept of assessing relative trade-offs in product attributes is known as Total Systems Effectiveness (TSE) and is needed to guide both product choice decisions at the country level and prioritisation of vaccine product related research and development (R&D), which may be at the country, regional or global level.

**Table 1:** Examples of vaccine product innovations with perceived benefit for LMIC immunisation programmes that are either facing slow development or limited country uptake.

Innovation	Perceived programmatic benefit for vaccine delivery	Status of product development or uptake	Reason for slow development/low uptake
Microarray patches (MAPs) [6], [7]	Single dose, ease of use, safety (needle-free and no reconstitution required), acceptability (potentially pain-free), potential for administration by community health workers or caregivers, potential for dose sparing	Early stage clinical studies for influenza and Hepatitis B vaccines; preclinical studies for vaccines recommended in EPI.	Poorly defined value proposition for EPI vaccines: unclear use case or demand forecast; significant investment needed for commercial manufacturing; acceptable/affordable price for EPI vaccines unknown, no clear market or procurement commitment.
Disposable syringe jet injectors (DSJIs) [8]	Single dose, safety (needle free and no reconstitution required), can be used with current liquid and lyophilised vaccine presentations, acceptability (potentially pain-free), potential for dose sparing	Intramuscular and intradermal devices WHO prequalified in 2004 and 2018, respectively. Clinical data have been generated with various vaccines including measles, mumps, rubella (MMR), inactivated poliovirus, BCG, HPV, as well as vaccines in development for dengue and Zika	Concerns about use case and programmatic fit, price and delivery cost.
Preformed compact pre-filled autodisable devices (cPADs), [9]	Pre-filled single dose, ease of use, autodisable, potentially suitable for use by lesser trained vaccinators, potential for improved acceptability	One preformed CPAD (Uniject ™) is currently available on the market. Available for hepatitis B vaccine, but only one vaccine manufacturer, and product is not available globally.	Initial issues with manufacturing process consistency and price. Unclear willingness-to-pay, significant investment needed for commercial manufacturing, no current procurement commitment/mechanism.
Vaccines qualified for controlled temperature chain (CTC) use [10]	Vaccine delivery cost efficiencies, ability to reach remote populations, reduced health worker burden	Available for some cholera, HPV, and meningitis A vaccines. Limited uptake; slow re-labelling of thermostable vaccines.	Limited quantitative evidence on added value for countries; may need more sensitisation of country stakeholders; not all vaccines are CTC compatible; manufacturers poorly informed of country preferences

The premise of TSE was conceived in 2012 by WHO, BMGF and PATH, in recognition of the need for improved tools and approaches to look beyond vaccine price per dose and to assess systems costs, as well as other factors such as immunisation coverage and equity, especially when evaluating products that incorporate potentially game-changing innovations [2]. This original TSE concept is one of the components of the Gavi healthy markets framework [3], which constitutes a broader array of strategies to address market failures of vaccines for LMICs. However, it was realised early on that a comprehensive framework to truly assess the TSE component of the healthy market framework was lacking. In 2014, the Bill & Melinda Gates Foundation began work on a TSE analytical tool and in 2016 they led a collaborative effort to develop a TSE framework with WHO, Gavi, PATH, and UNICEF. Complementary tools were also developed, such as PATH’s Vaccine Technology Impact Assessment (V-TIA) tool [4], which estimated full systems cost and considered the trade-offs between cost, health impact, safety and coverage and equity through a dashboard. In 2018, Gavi, WHO, UNICEF, PATH and BMGF built on the TSE indicators to develop an analytical evaluation framework to assess vaccine product innovations through the newly launched Vaccine Innovation Prioritisation Strategy (VIPS) initiative. VIPS aims to prioritise and drive vaccine product innovations to better meet country needs and support Alliance coverage and equity goals [5]. WHO also advanced a specific TSE approach (hereinafter referred to as WHO TSE) that primarily focused on incorporating the country perspective into immunisation decision-making, in order to address the needs of member states.

The aim of this paper is to present the original concept of TSE, formulated by BMGF, Gavi, PATH, UNICEF and WHO, and to introduce the WHO TSE approach and the experience from initial piloting in countries, including use of TSE approach to guide rotavirus vaccine introduction and switch recommendations. The paper outlines learnings to inform and guide further development of TSE to optimise decision making at the country level, as well as efforts to ensure future innovations have better prospects of being adopted and scaled up in countries where they could have significant impact on vaccine coverage and equity.

## 2 The original TSE concept

The original TSE approach hypothesises that market failures of innovative vaccine products and technologies (hereinafter referred to as “innovations”), are due to asymmetric information between countries and innovators: country-level product selection decisions may often be influenced by purchase price and supply availability, without a comprehensive review of the impact that product features could have on the broader health system, particularly in terms of vaccine delivery cost savings, programme efficiencies or equitable coverage. At the global level, since innovative products often have a higher price point to recoup investment in product development, price-based decisions create significant risk for developers, who need to ensure there is sufficient demand and willingness to pay. Furthermore, without clarity on preferred product characteristics for new products from country programmes and procurement agencies, it is challenging to derive appropriate product specifications, creating reluctance for vaccine and technology developers to invest in innovation. Examples of recent vaccine delivery innovations that have not achieved the anticipated uptake or impact, are shown in Table 1.

The original TSE is envisioned as a method to evaluate and compare different vaccine products from a systems perspective, in order to support stakeholders across the product development continuum (including LMIC immunisation programmes), to analyse trade-offs between different products. The approach is conceptualised as an end-to-end analytical framework comprising a common and comprehensive set of elements to analyse trade-offs between innovative product attributes. It is a multi-criteria decision analysis (MCDA) approach, as a structured, explicit way to take account of multiple factors during decision-making [11]. The framework is composed of five components (or criteria) and a series of indicators within each component was developed to quantify these trade-offs (Table 2).

**Table 2:** Components of the original TSE framework.

TSE component		Description
	Health impact	To what extent does the vaccine presentation protect against disease? *Indicator: efficacy; effectiveness; factors affecting potency and timeliness of vaccination; duration of protection*
Impact	Coverage	How does the vaccine presentation affect the proportion of the target population receiving the full vaccination schedule? Could the vaccine presentation decrease equity gaps in immunisation? *Indicator: incremental coverage improvement (indicated by: vaccination schedule, storage requirements, administration requirements, acceptability, doses per container)*
	Safety	Does the vaccine presentation have a lower safety risk? *Indicator: adverse events following immunisation (AEFI); risk of programmatic error (incorrect preparation, contamination, incorrect delivery, needle-stick injury)*
Cost	Commodity cost	What is the cost of the vaccine and supplies, for complete vaccination factoring in wastage? *Indicator: vaccine cost, delivery technology cost, safety box cost*
	Vaccine delivery cost	What are the operational costs to deliver the vaccine? *Indicator: storage cost, transport cost, administration cost, waste disposal cost, monitoring and evaluation, introduction cost (including training, storage expansion, social mobilisation/communication)*

## 3 The WHO TSE framework

**Fig. 1:**
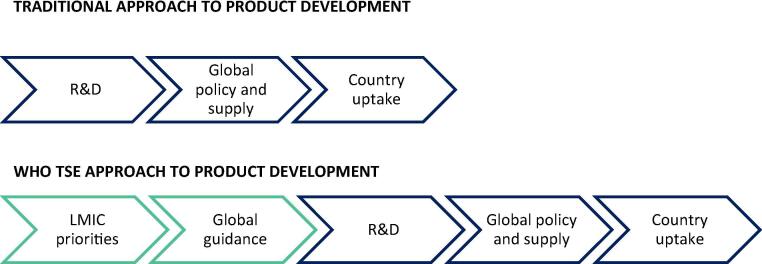
The concept of WHO Total Systems Effectiveness (TSE) is to improve the alignment of global R&D with the needs and preferences articulated by LMIC immunisation programmes, in order to accelerate development and uptake of vaccines.

From WHO’s perspective, TSE has an important role in shaping the priorities for upstream development according to the factors determining country uptake of vaccine products. It was envisioned that countries could use the TSE framework with their own local data to examine trade-offs between existing or pipeline products. This would serve both country decision-making and allow vaccine development stakeholders to model the impact of different product attributes and price points. In doing so, WHO’s vision for TSE is to change the paradigm of product development such that country preferences and demand are well-articulated to drive investment decision-making. By clearly determining the value that a new product could offer to country and global decision-makers early in the development process, TSE could create a pull for innovation (Fig. 1). The framework that emerged from piloting the TSE concept with country level stakeholders is referred to as the WHO TSE framework.

## 4 Methodology to pilot the WHO TSE framework

In 2018, in order to explore whether TSE would be a useful tool for WHO member states, WHO began a country-centred project, to further develop and test the concept of TSE in LMICs, using Rotavirus vaccine products as a test case. The goal was to determine the utility of using a multi-criteria decision-making approach with country-level data for comparing different products of the same vaccine, each with varying attributes and characteristics. The pilot tested the following hypotheses, which had been developed jointly by the original TSE partners, based on their experience:

• **Hypothesis 1:** Decision-making processes in LMICs do not currently take a structured systems perspective for immunisation product evaluation and selection. There is interest from country policymakers to introduce such a systems perspective.
• **Hypothesis 2:** Vaccine development stakeholders have a limited understanding of LMIC needs, preferences and demand, which disincentivises investment in products tailored for use in LMICs and leads to misalignment between products in the pipeline and LMIC needs.
• **Hypothesis 3:** A common framework to evaluate vaccine products, used by immunisation programme, global policy/procurement and vaccine development stakeholders, could align R&D priorities and global supply/procurement with country priorities and preferences.

To test the first hypothesis, consultations were held in pilot countries on the existing process for product selection, in order to evaluate whether TSE could be a helpful approach in their setting. Three initial pilot countries (Indonesia, Mali, and Thailand) were selected, following a call for interest to WHO regional and country offices. An effort was made to ensure geographical diversity and to include countries with different income levels, immunisation programme maturity, status of rotavirus introduction and strength of policy setting (Table 3). In each pilot country, 1–2-day workshops were conducted with stakeholders involved in the vaccine product selection policy process. Since product selection processes vary between countries, a core team in each pilot country identified the stakeholders. The core team was composed of stakeholders from the national immunisation programme (Mali), WHO country office (Mali and Indonesia), the national immunisation technical advisory group (NITAG) (Indonesia), and/or the health technology assessment (HTA) agency, which conducts comparisons of health interventions (Thailand). Workshops consisted of a review of the existing product selection process, followed by an introduction to the TSE concept and orientation to an Excel-based TSE model for product selection. At the close of the workshop, participant feedback was collected through discussion and an anonymous survey on interest to use the TSE approach for product selection. Further evaluation was conducted by the country core team on the advantages and disadvantages of the TSE approach and shared with WHO.

**Table 3:** Profile of countries included in the pilot to test hypothesis 1.

Pilot country	Region	Income status (2018)*	Gavi financing status	Policy setting maturity**	Rotavirus vaccine in NIP
Indonesia	South-East Asia	LMIC	Fully self-financing	6	No
Mali	West Africa	LIC	Initial self-financing	3	Yes
Thailand	South-East Asia	UMIC	Not Gavi eligible	6	No

**LIC –** low income country, **LMIC –** lower middle income country, **NIP –** National Immunisation Programme, **UMIC –** upper middle income country.

* According to World Bank classification.

** Measured according to the WHO/UNICEF Joint Reporting Form national immunisation technical advisory group (NITAG) indicator, in which 0 is the minimum and 6 is the maximum score.

To test the second hypothesis, a workshop was held in Thailand (August 2018) with representation from local vaccine manufacturers in Indonesia, Thailand and Vietnam, as well as stakeholders from immunisation programmes, academia and international organisations from the region. During the workshop, participants discussed the challenges faced by vaccine manufacturers, their priorities for investment, and their current interactions with immunisation programme stakeholders. At the close of the workshop, participants gave written anonymous feedback on the utility of the TSE approach and whether it would be informative for R&D. The methods and results from this workshop are fully detailed in another paper [12]. Findings from the workshop were supplemented by discussions with vaccine development stakeholders at the global level.

**Fig. 2:**
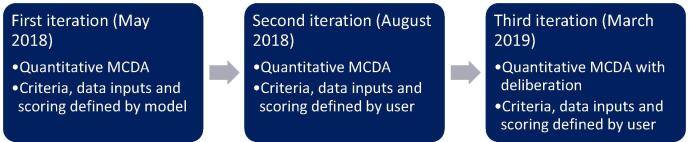
Summary of the three iterations of the WHO TSE model for rotavirus product selection.

To address the third hypothesis, an Excel-based model was developed for comparison of both existing and pipeline vaccine products, according to the indicators identified in Table 2. Rotavirus vaccine products were selected as a test case, since there are a variety of products on the market and pipeline presentations with diverse characteristics [13]. The initial intention was to train the country core teams in Indonesia, Mali and Thailand to use the model, following which they would populate the model with country-specific data and present the outcomes to national product selection stakeholders (as in hypothesis 1) and vaccine development stakeholders (as in hypothesis 2) to identify whether the model simultaneously addressed the needs of both groups. However, since the initial Excel model did not have traction with national immunisation programme stakeholders, the model (and the way in which it was tested) evolved throughout the pilot and was tested as three iterations (Fig. 2).

**First iteration (May 2018):** An Excel-based MCDA model for rotavirus vaccine product selection was developed based on the pre-defined components and indicators specified in the original TSE framework (Table 2). The model can be found in supplement 1. For the purpose of the pilot, the model was pre-populated with data on existing and pipeline rotavirus vaccine characteristics and the country users entered country-specific data. The model incorporated consideration of equity by allowing users to consider different programmatic needs of sub-national groups and settings (e.g. by wealth quintile or geographic region) and allowed modelling of scenarios to introduce different products for different sub-populations within a country. Where appropriate, existing models were used as inputs for each component of the model, including UNIVAC (for health impact) [14], V-TIA (for commodity and delivery cost) [4], and C4P (for commodity and delivery cost) [15]. Using quantitative MCDA, the model calculated a score between 0 and 100 per framework component and summed the scores across all components to give a total score per vaccine product. The model output was an overall ranking of rotavirus vaccine products (from highest to lowest total score), together with a decision matrix showing the rationale for the ranking [16]. As a final step, the characteristics of the pipeline products could then be modified to identify the profile thresholds for which the pipeline products would rank above the existing products (for example, to identify whether an increase in efficacy or reduction in cold chain volume would alter the ranking of the pipeline product). During the pilot, this model was populated with national data in Thailand and Indonesia, and the outputs were reviewed as part of the workshops conducted for hypotheses 1 and 2.

**Second iteration (August 2018):** In response to feedback that the first iteration of the model was too complex and inflexible (see results), an alternative Excel-based model was developed, using the MCDA component of the Public Health England prioritisation framework [17]. This revised model allowed the users to define criteria and data needs, as well as supporting country teams to define their own scoring scale. The model can be found in supplement 2. Workshops were held in Mali and Indonesia (one day), with the stakeholders outlined in methods for hypothesis 1, to conduct a test case to select between existing rotavirus vaccines and a hypothetical next generation injectable rotavirus vaccine. At the close of the workshop, feedback was collected on utility for national immunisation decisions through a discussion led by the country core team. The WHO HQ team then extracted and analysed information from the exercise, for the original TSE partners to review whether the outputs from the model would be informative for vaccine research and development.

**Third iteration (March 2019):** Incorporating feedback on the second iteration from core teams in Mali and Indonesia, the model was further revised to adopt a procedural approach, in which the tool was structured around five key steps in a recommendation process. The revised model incorporated guidance on stakeholder and criteria selection, as well as analytics to support the interpretation of the total MCDA scores generated by the model. See supplement 3. To test this model, one-day workshops were held during regional meetings in Abidjan and Bujumbura with the NITAG chair, EPI manager, and WHO country office focal point of Benin, Burkina Faso, Central African Republic, Côte d’Ivoire, Democratic Republic of the Congo, Ghana and Nigeria. These countries were identified by the regional office as countries with pending rotavirus switch or introduction decisions. Workshops opened with training on existing rotavirus vaccine products for consideration, but otherwise followed the same format as for the second iteration of the model, with global level analysis of the data for R&D. This model was also tested in full by the Mali NITAG to select between available HPV vaccine products, following which the NITAG, EPI and country office evaluated the utility of the model and presented their feedback to the WHO immunization and vaccines related implementation research advisory committee (IVIR-AC) in September 2019.

## 5 Results

The WHO TSE pilot found significant interest from both immunisation programme and vaccine development stakeholders to implement a systematic approach to priority-setting for national immunisation programmes and for vaccine R&D and supply. Table 4 summarises the findings across all hypotheses.

**Table 4:** Summary of original assumptions and results from the WHO TSE pilot. Whilst the principles of all hypotheses were found to hold true, it was found that for both national immunisation programme and R&D decisions, there exists a gap in applying country context and values for product evaluation and priority-setting.

	Hypothesis	Finding
1	Decision-making processes in LMICs do not currently take a structured systems perspective for immunisation product evaluation and selection. There is interest from country policymakers to introduce such a systems perspective.	LMIC policymakers would appreciate support to consider multiple factors during decision-making (trade-offs), for a wide range of policy and programme questions (not just product selection). There is particular interest to identify and incorporate country-specific criteria.
2	Vaccine development stakeholders have a limited understanding of LMIC needs, preferences and demand, which disincentivises investment in products tailored for use in LMICs and leads to misalignment between products in the pipeline and LMIC needs.	Country and global vaccine development stakeholders would benefit from a better understanding of country preferences, as no mechanism currently exists to collect country input for product development on a systematic basis.
3	A common framework to evaluate vaccine products, used by immunisation programme, global policy/procurement and vaccine development stakeholders, could align R&D priorities and global supply/procurement with country priorities and preferences.	Preferences for current and future products may differ. However, strengthening structured processes for LMICs to determine priorities and values for their context allows better articulation of needs from pipeline products.

### Hypothesis 1: there is a need to take a systems perspective in country product evaluation and selection

In Indonesia, the workshop included participants from the EPI, planning unit within Ministry of Health, national regulatory authority, NITAG (and health economics working group), SEAR-RITAG, BioFarma, WHO country office and Gavi. It was discussed that Indonesia already incorporates economic and health systems considerations for decision-making, but that a more systematic approach to take account of various variables is needed. Especially after the decentralisation reform has been implemented, an approach to include sub-national analysis (at least at the provincial level) will become important, incorporating issues such as equity and different local government capacity, but there will be issues with data availability. It was highlighted that NITAG recommendations are often not implemented due to lack of budget. The group therefore saw broader applicability of, and need for, the TSE approach for prioritisation, including to support EPI to prioritise which new vaccine introduction recommendations to implement, to prioritise regions or districts if there are insufficient resources for national introduction, and to compare vaccination with other prevention and control measures to advocate for increased budget allocation. The NITAG also highlighted that TSE could help to direct the research agenda by identifying the most important data gaps that must be addressed to inform decision-making.

The Thai workshop was attended by representatives from the national essential list of medicines (NELM), NITAG, HTA agency, national vaccine institute, national regulatory authority, and representatives from pharmaceutical companies. It was felt that Thailand already had well-established processes to combine multi-criteria decision analysis with economic evaluations for vaccine introduction and procurement decisions, but that a TSE approach could potentially support discussions on combination vaccines or medical devices.

The workshop in Mali included representation from the Ministry of Health (EPI, surveillance, budget, communications), Ministry of Finance, NITAG, and members of the inter-agency coordination committee (ICC). It was discussed that the existing process for new vaccine introduction and product selection is mainly based on burden of disease data and product availability. A structured process to incorporate multiple criteria for decisions – especially if these could be country-specific – would be beneficial, especially in supporting the newly established NITAG.

### Hypothesis 2: vaccine developers need a better understanding of country needs

Local and global vaccine development stakeholders in the public and private sector all agreed that priority-setting and definition of target product profiles lacks input and perspective from country immunisation programmes, even though it is essential for informing R&D priorities. However, it was also highlighted that national immunisation programme stakeholders may have limited time or interest to engage in dialogue on development of vaccines that are several years from licensure and may never reach the market. Local manufacturers often have dialogue with the immunisation programme, but these discussions can be ad-hoc, and it was highlighted that an understanding of neighbouring country needs would support local manufacturers to expand their market. Whilst an improved understanding of the criteria used in country product selection would be useful, it was highlighted that TSE brings most value in understanding the perspective of different stakeholders at the country level and the rationale for their preferences.

### Hypothesis 3: a common framework to evaluate vaccine products could align better align priorities between R&D and country stakeholders

Implementing a common framework across current programme decision-making and R&D prioritisation could allow programme stakeholders to better define their values and priorities to improve consistency of input to R&D so that country-specific needs are considered. However, it was consistently found that there is a need to expand on the deliberative component of decision-making: for country decisions, this includes guidance on stakeholder selection, identifying country-specific priorities, and using the evidence to come to a recommendation (by promoting a shared understanding of the pros and cons of each option among the group); for vaccine development this includes understanding the rationale for preferences from a range of stakeholders at the country level.

**First iteration:** stakeholders in Indonesia and Thailand indicated that the Excel model required very specific data and did not incorporate all immunisation programme priorities. For example, stakeholders wanted to incorporate cost-effectiveness analysis, budget impact analysis, supply considerations (diversity of manufacturers, availability, local manufacture), global and national policy, and, in Indonesia, whether the product is halal. It was highlighted that the model needs to be modifiable by country stakeholders. In terms of the link to R&D, there was concern about combining current and future products in a common model, since preferences for existing and future products may differ, and certain innovations will only be useful for countries if they are applied across multiple vaccines. It is essential to clearly define the alternatives being compared (in terms of the target population and vaccine delivery approaches for products), since preferences will depend on how the product would be introduced and used.

**Second iteration:** in the workshops in Mali and Indonesia, immunisation programme stakeholders welcomed the revised model as a consultative process to simultaneously consider many country-specific issues and to have the flexibility to use data that is available to the country. However, both countries requested more guidance around the process to identify and engage stakeholders, and to select criteria. In Indonesia, this was considered particularly important given decentralisation in healthcare decision-making. When analysing the results from the workshop for R&D, it was possible to extract preferred criteria and relative importance for a specific decision question, but it proved challenging to define specific minimal targets that products should meet to ensure country uptake.

**Third iteration:** West and Central African stakeholders highlighted the value of the model in facilitating county-specific discussions, encouraging transparent documentation, and in considering data uncertainty in recommendations. In Benin and DRC, the TSE approach subsequently helped to structure rotavirus product choice recommendations. Feedback from the Mali NITAG was that the tool was easy to use and allowed a consensual and scientific approach to decision-making, that can incorporate many criteria and stakeholder perspectives. The NITAG particularly appreciated the possibility to examine the implications of data uncertainty and to automatically generate a summary report, but noted that the model could take greater account of immunisation programme constraints, such as cold chain and human resources capacity. Regarding the link to R&D, certain stakeholders voiced hesitancy around sharing the completed version of the tool with global stakeholders. The Mali NITAG noted that with this model, another forum would be needed to communicate needs to vaccine developers: although the country faces many challenges with the existing BCG presentation, for example, if the country does not use the model for a BCG policy question, they have no mechanism by which to signal this need.

## 6 Discussion

Whilst the challenge of identifying which innovations to invest in is not new, assessing the value of innovation is becoming increasingly relevant as R&D funding is constrained and as countries strive to close equity gaps through raising stagnating vaccine coverage. Each year 20 million children under one year of age do not receive their full series of recommended vaccinations, with most of these children at highest risk of disease and belonging to the lowest socio-economic groups [18], [19]. More than ever, countries need to be equipped to consider whether to invest in innovations that may improve coverage and equity, but at the expense of a higher price tag or disruption to the status quo for vaccine delivery. Equally, innovators need to understand the types of products in which to invest to meet market demand. This is especially relevant in the COVID-19 context, as countries are faced with decisions around which immunisation activities to prioritise and which populations to target with a potential vaccine, while global partners have a role to ensure that any COVID-19 vaccine that does come to market is suitable for roll-out in LMIC settings.

Pilot countries and vaccine developers welcomed TSE as a framework to allow a structured, systems-based comparison of products. It was concluded that any tools and processes to support country decision-making must have adaptability, in terms of alignment with existing country policy processes, applicability across policy questions (not confined to product selection), flexibility to select criteria that reflect country and social values, and the ability to function across countries with different data availability and analytical capacity. Furthermore, policymakers highlighted the need for guidance through the deliberative process to come to a consensus-based recommendation, including stakeholder identification, incorporating country programme needs into discussions, and guiding discussions so that the committee (for example, NITAG, inter-agency coordinating committee (ICC), or EPI planning unit) has a shared understanding of the decision question, evidence, and data limitations in coming to a recommendation. Leadership by Ministries of Health is essential to convene key stakeholders and ensure credibility of the approach. In order for this to occur, the tools and processes developed should be streamlined and intuitive for the end-user, as well as being applicable to policy questions relevant to the immunisation programme and amenable to the existing HTA and immunisation programme policy processes in a country. The ISPOR (International Society for Pharmacoeconomics and Outcomes Research) report on good practise in HTA makes a distinction between ‘assessment’ (synthesising evidence) and ‘contextualisation’ (using evidence) [20]. This pilot identified a gap for processes and tools to support contextualisation in immunisation decision-making, for both country programme and vaccine development priority-setting. A number of initiatives already exist to support the assessment stage of national immunisation programme policy recommendations, most notably UNIVAC (formerly ProVac), PriorityVax, and tools for NITAG strengthening. However, in line with previous findings for health sector priority-setting and reviews of immunisation policy setting in LMICs, the pilot confirmed a need to support countries to use the evidence to come to a recommendation that is founded on their specific priorities and programme needs [21], [22], [23].

This is in line with calls to recognise the perspectives of country-level stakeholders and the decision context when considering value of vaccine products under development [24]. There is currently no established platform to engage with immunisation programmes to assess the extent to which pipeline products could address their needs or to solicit preferences for future products and how they would be used. TSE could strengthen communication to R&D stakeholders on the needs, demands and preferences from LMIC immunisation programmes for future innovation. To be successful, TSE must incorporate a mechanism to share this information with vaccine development stakeholders, and to monitor changes in preferences over time.

The premise of supporting countries to take context-specific and evidence-based decisions is consistent with the principles of the Immunisation Agenda 2030, which emphasise the importance of decisions being country-owned and data-enabled [24]. To be successful, it is essential that TSE concepts be embedded within immunisation decision-making processes and legislative frameworks, and the WHO approach is designed to that end. At the country level, this is likely to include articulation of where TSE tools fit within existing decision-making infrastructure, both within the immunisation programme and broader health sector priority-setting mechanisms such as health technology assessment (HTA) and benefits package selection. At the global level, TSE must play an integral role in the development of global guidance for vaccine development and supply, in order to place the country perspective at the centre of the product development continuum.

There are a number of questions regarding the feasibility of implementing the WHO TSE tool for use by country level stakeholders. In the studies to date, the WHO TSE tool has elicited interest within the bounds of the pilot; whether countries would adopt and provide the necessary resources to continuously use the tool outside a pilot setting remains to be seen. TSE can offer benefit, especially with the increasing appreciation that ‘one size does not fit all’ and differentiated vaccine delivery strategies may be needed at the sub-national level to meet the variable health system environments and infrastructure within countries. The WHO TSE tool could play a role in empowering LMICs to identify which combination of product presentations and approaches will best meet their needs to optimise the immunisation programme and reduce inequities in immunisation coverage. Moreover, through implementing TSE, WHO will be positioning LMICs to influence vaccine innovation priorities and R&D, which is essential to ensure that new vaccine products overcome immunisation programme challenges and optimise the public health benefit of immunisation programmes.

## 7 Conclusion

In our study in 10 LMICs, the original TSE approach to use pre-determined criteria for product evaluation (as outlined in Table 2) was not found to be acceptable to country stakeholders, who are key to understanding the value of innovations. Instead, inclusive, workshop-style approaches may better foster discussion around country needs, expanding the extent to which these are addressed by pipeline products, and clarifying acceptable trade-offs between product characteristics from the perspective of LMIC stakeholders. Since the WHO TSE framework criteria are not pre-determined, building country capacity to articulate their values and criteria for decision-making will improve the consistency and credibility of the outputs from such workshops. Over the next decade and beyond, it is expected that there will be an ever-growing range of innovations available to countries. This will only increase the complexity of making informed decisions, particularly in the absence of a structured process to weigh the trade-offs of each option in a given country context. Acknowledging this need, WHO has continued the piloting and optimisation of the WHO TSE approach and has rebranded this as CAPACITI (Country-led assessment for prioritisation on immunisation). The lessons from the TSE pilot with rotavirus vaccine will inform further development and expansion of the TSE concept and related tools, with a vision to build towards a framework for countries and product developers to evaluate portfolios of products and innovations, such that innovations are not considered on an individual vaccine basis, but as a comprehensive set of approaches for countries to achieve their immunisation targets.

## Declaration of Competing Interest

The authors declare that they have no known competing financial interests or personal relationships that could have appeared to influence the work reported in this paper.
